# The current epidemic status and prevention and control of bovine viral diarrhea virus in yaks in China

**DOI:** 10.3389/fcimb.2025.1647328

**Published:** 2025-09-09

**Authors:** Zhi Li, Yuan Han, Qing Yuan, Shuqin Wang, Changjiang Chen, Shandian Gao, Ru Meng

**Affiliations:** ^1^ Qinghai Provincial Key Laboratory of Pathogen Diagnosis for Animal Diseases and Green Technical Research for Prevention and Control, Academy of Animal Sciences and Veterinary Medicine, Qinghai University, Xining, China; ^2^ Xining Animal Disease Prevention and Control Center, Xining, China; ^3^ Huangyuan County Animal Husbandry and Veterinary Station, Xining, China; ^4^ Gansu Province Research Center for Basic Disciplines of Pathogen Biology, State Key Laboratory for Animal Disease Control and Prevention, Lanzhou Veterinary Research Institute, Chinese Academy of Agricultural Sciences, Lanzhou, China

**Keywords:** bovine viral diarrhea virus, yak, epidemic status, prevention and control, five-dimensional integration

## Abstract

In China, yaks are predominantly distributed across the Qinghai-Tibet Plateau and surrounding high-altitude regions, including Tibet, Qinghai, Sichuan, Gansu, Xinjiang and Yunnan. These animals serve as multifunctional resources for local herders, providing meat, dairy, hides, and wool, while also constituting a critical component of the industrial chain in high-altitude ecosystems. Recent epidemiological studies have demonstrated an increasing trend in bovine viral diarrhea virus (BVDV) infection rates among yak populations in provinces such as Gansu, Sichuan, Tibet, and Qinghai. This review not only summarizes the epidemiological status, distribution of viral sub-genotypes, and current prevention and control in yaks across various regions, but also proposes, for the first time, a systematic “Five-dimensional Integration” comprehensive prevention and control model for BVDV, including vaccine breakthrough, precise monitoring, dynamic early warning, population purification, and active prevention, which will provide directed insight for the prevention and control strategies of yaks infected by BVDV.

## 1 Introduction

The yak (*Bos grunniens*) is the only cattle species globally adapted to the alpine and hypoxic environment at altitudes of more than 3,000 meters. It is an iconic species of the Qinghai-Tibet Plateau. In 2021, the stock of yaks in China was approximately 16.3595 million, accounting for more than 94% of the global total, followed by neighboring countries such as Mongolia, Russia, India and Nepal. In China, the yaks are mainly distributed in Qinghai, Tibet, Sichuan, Gansu, Xinjiang, Yunnan and other provinces and regions, and there are more than 20 local and cultivated breeds such as the Datong yak and the Ashtan yak, which not only provide herders with various products such as meat, milk, fur and bones, but also serve as a mainstay industry for the economic development of the Qinghai-Tibet Plateau and its surrounding alpine pastoral areas. Moreover, the yaks also play an important role in maintaining the pasture ecosystem and protecting agricultural biodiversity (Han and Liang, 2024; Huang et al., 2023). The yak breeding still faces significant challenges in epidemic prevention and control due to multiple factors such as high altitude and cold climate constraints, traditional grazing-based management practices, weak infrastructure in pastoral areas, difficulties in covering epidemic disease monitoring, and limitations of drugs and vaccines. Current epidemiological data indicate that yak diseases are seasonal and regional, prone to bacterial infections caused by *Pasterella* spp. and *Escherichia coli*, as well as viral infections including bovine coronavirus, bovine parainfluenza virus, rotavirus, infectious bovine rhinotracheitis virus, and bovine viral diarrhea virus (Du, 2020); and there are various ectoparasites (such as ticks and mites) and endoparasites (such as flukes and nematodes) (Lei et al., 2016). In addition, the threat of echinococcosis can also threaten public health security (Zhuo, 2024). In recent years, bovine viral diarrhea virus has attracted attention in Gansu, Sichuan, Tibet, Qinghai and other provinces and regions where the infection rate of yaks is increasing. This review summarizes the epidemiological status of BVDV in yaks across different regions, the distribution characteristics of viral subtypes, and the current prevention and control status. Furthermore, the paper describes the prevention and control measures to provide references for the prevention and control of BVDV in yaks.

## 2 Etiology and pathogenic characteristics of BVDV

BVDV is an enveloped, positive-strand RNA virus belonging to the genus *Pestivirus* within the family *Flaviviridae*. It is homologous to other important animal viruses such as classical swine fever virus (CSFV) and border disease virus (BDV). The C protein, E^rns^, E1 and E2 encoded by the BVDV genomic RNA are structural proteins that bind to lipid bilayer membranes to form 40–60 nm viral particles (Al-Kubati et al., 2021). Virus particles maintain stability at pH 5.7–9.3 but are susceptible to organic solvents and detergents. Temperatures above 40°C temperatures compromise viral viability. BVDV can infect cloven-hoofed livestock such as cattle of all ages, sheep, goats, pigs, and camels, as well as various cloven-hoofed wild animals including African buffalo, oryx, Canadian bison (*Bison bison bison*), Pudu (*Pudu puda*), alpacas, camelids, antelope, roan antelope, roe deer, chamois and pronghorn antelope (Vilcek and Nettleton, 2006; Passler and Walz, 2010). According to viral culture characteristics in cells, BVDV can be divided into two biotypes: non-cytopathogenic (NCP) and cytopathogenic (CP). The NCP-biotype BVDV is the main cause of acute infections and can be transmitted through various body fluids, including nasal secretions, urine, and milk. When cattle are infected with BVDV, obvious clinical symptoms such as significant fever, persistent diarrhea, bloody or mucoid stools, mucosal ulcers, abortion, and reproductive disorders may occur. These symptoms can affect production performance or cause cattle deaths, leading to economic losses. Therefore, the World Organization for Animal Health (WOAH) lists bovine viral diarrhea (BVD) as a notifiable infectious disease in cattle, while China classifies it as a Class III animal disease. Previous studies showed that transplacental infection of 40- to 120-day-old fetuses with NCP-biotype BVDV can lead to abortion, immune tolerance, and the production of persistently infected cows, and that utero infection with BVDV during the period of organogenesis and immune system development in bovine fetuses of 100- to 150-day can lead to congenital malformations (Grooms, 2004). BVDV infection in cattle typically leads to secondary infections with bovine herpesvirus type 1, bovine parainfluenza virus type 3, bovine respiratory syncytial virus, infectious bovine rhinotracheitis virus, and bovine respiratory coronavirus, causing bovine respiratory disease complex (BRDC) and affecting production performance, whereas persistent infections and reproductive disorders resulting from infection of pregnant females by NCP strains can directly cause economic losses. In addition, persistently infected cows not only seriously threaten their herds with massive lifelong viral shedding, but also the NCP-biotype BVDV in their bodies can also mutate into the CP biotype, leading to the development of mucosal disease (Lanyon et al., 2014).

The acute course of BVDV infection in yaks lasts 5~7 days, while the chronic course can extend to half a month or more. The main clinical manifestations include pyrexia (above 40°C), watery or hemorrhagic diarrhea, oral ulcers, salivation, and cessation of rumination. Necropsy reveals congestion and hemorrhage in the digestive tract, along with intestinal mucosal detachment and hepatomegaly. In pregnant dams, abortion and infertility may occur. In cases involving the respiratory system, serous to purulent nasal discharge is observed. The main pathological findings include congestion, hemorrhage, and necrosis in the digestive tract, with some cases also showing mucopurulent nasal discharge or caseous plugs obstructing the airways (Liu, 1984; Ji et al., 1992; Li and Jiang, 2007).

## 3 Genetic diversity of BVDV

Currently, the International Committee for the Classification of Viruses classifies bovine viral diarrhea viruses into three species: Pestivirus bovis (BVDV-1), Pestivirus tauri (BVDV-2), Pestivirus brazilense (HoBiPeV) (https://ictv.global/report/chapter/flaviviridaeport/flaviviridaeport/flaviviridae/pestivirus) (Postel et al., 2021). Currently, BVDV genotyping and sub-genotyping rely on sequencing the 5’ UTR, N^pro^ gene, and E2 glycoprotein coding region, classifying strains into BVDV-1 (a-x), BVDV-2 (a-e), and HobiPeV (a-e) (Deng et al., 2020; Giangaspero et al., 2008; de Oliveira et al., 2022; Kalaiyarasu et al., 2022; Mucellini et al., 2023). In terms of global distribution, BVDV exhibits high genetic diversity in Europe and Asia (such as Turkey and China), and the genetic variation of the virus caused by gene recombination of different genetic subtypes cannot be ignored. BVDV-1 is widely prevalent globally and mainly causes respiratory disease, enteritis, and embryonic infection in cattle. BVDV-2 has been reported in the United States, Brazil, Argentina, countries such as Germany, Belgium, France, the United Kingdom, Austria, Slovakia, China and Turkey. BVDV-2 induces clinical disease in cattle characterized by characterized by hemorrhage, pneumonia, fever, hemorrhagic mucoid diarrhea, dyspnea, and a relatively high mortality rate, but the prevalence of BVDV-2 is significantly lower than that of BVDV-1 is significantly lower than that of BVDV-1 (Yesilbag et al., 2017). HoBiPeV has been detected in cattle herds in countries in South America, Europe, Africa and Asia including Brazil, India, China, and Thailand since 2004 and was reported to be associated with severe respiratory disease in Italian cattle herds in 2010 (Decaro et al., 2012; Liu et al., 2012; Bauermann et al., 2013; Afify et al., 2022; Kalaiyarasu et al., 2022).

The earliest detailed record of BVDV research in China is in 1983, when the virus strain was first isolated (Li et al., 1983). Early epidemiological data showed that the BVDV antibody prevalence of BVDV in cattle herds across China was less than 20% in the early 1990s, which was relatively low (Zheng et al., 1991). However, by the end of the 1990s, the positive rates of BVDV antibodies in yellow cattle and yaks in the northwestern region reached 30.8-55.95% (Gao et al., 1999). Recent systematic analyses of dairy cattle populations in China have indicated that the seropositive rate of BVDV is 57.0%, and the positive rate of viral RNA is 27.1%, which was mainly concentrated in regions with developed animal husbandry (Ran et al., 2019). Molecular epidemiological data showed that there are numerous genotypes of BVDV in China, including BVDV-1a~BVDV-1d, BVDV-1m~BVDV-1q, BVDV-1u~BVDV-1w, BVDV-2a, BVDV-2b, BVDV-3 (HobiPeV) and so on (Li et al., 2024).

## 4 Prevalence of BVDV in yaks

**Figure 1:**
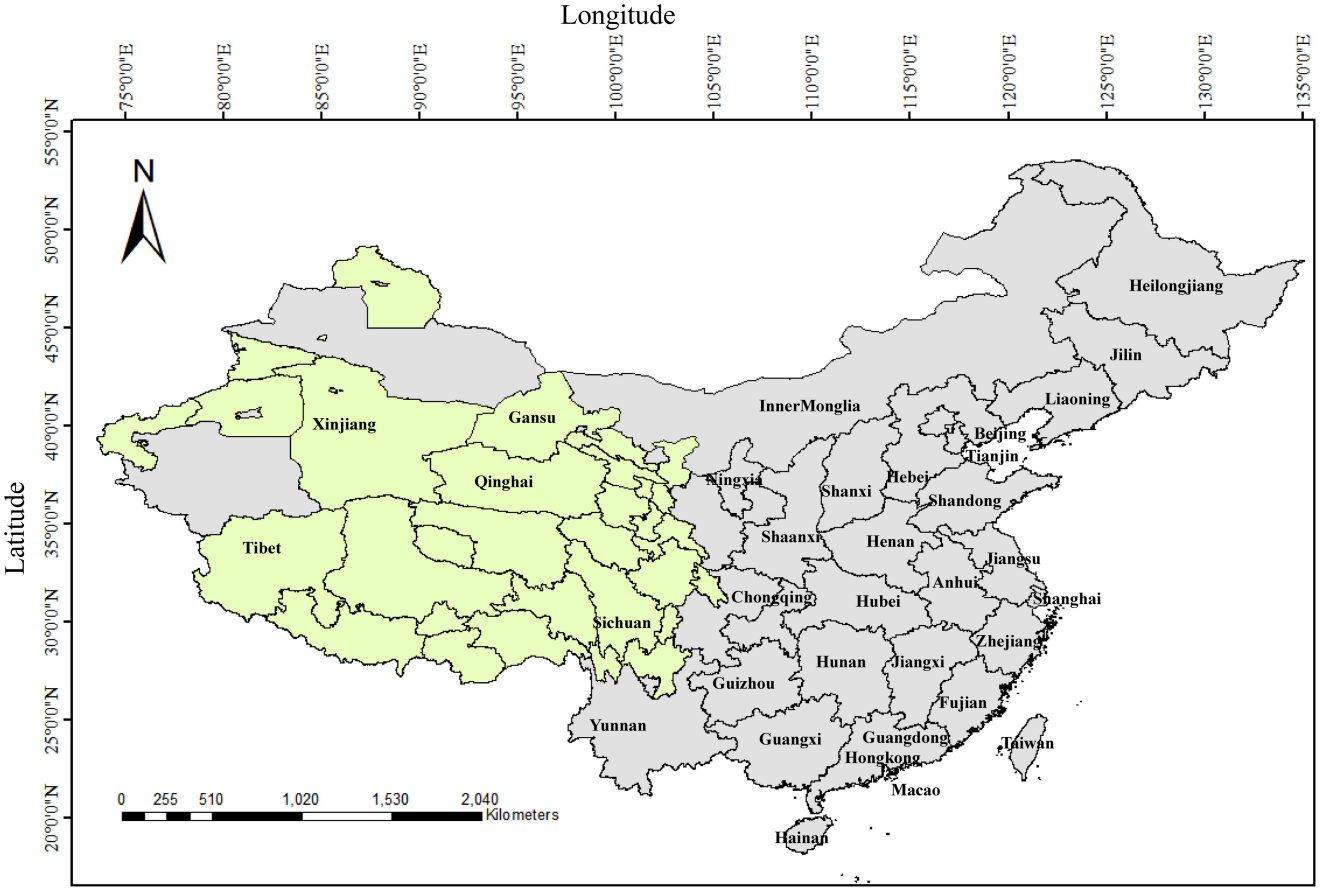
The geographic distribution of yaks in China. Olive green represents the regions of distribution for yaks in China.

China has the largest yak population in the world, accounting for more than 94% of the global total, and is mainly distributed in the provinces and regions of Qinghai, Tibet, Sichuan, Gansu, Xinjiang and Yunnan, with Qinghai accounting for 37.57% (6,145,800) of the national herd, followed by Sichuan at 25.82% (4,224,200), and the Tibet Autonomous Region at 25.59% (4,186,700) (Han and Liang, 2024) (**Figure 1**). Since the 1980s, bovine viral diarrhea has been recognized as an important viral disease closely related to yak production and has attracted attention. Until now, researchers have performed detailed studies on bovine viral diarrhea in yaks in Qinghai, Tibet, Sichuan and Gansu about etiological and serological aspects, but there is little epidemiological information on bovine viral diarrhea in yaks in Yunnan and Xinjiang. Since 1980, studies on the “diarrhea disease” of yaks in northwest China have shown that the disease occurs most frequently from April to September, coinciding with intensive grazing period; and the incidence is highest in adult yaks over four years of age, and yaks are more susceptible than Pian cattle (a hybrid offspring from crossbreeding between a yak and Chinese yellow cattle) and other breeds, and these infections are frequently complicated by secondary pathogens such as *Salmonella* and other intestinal pathogens, which exacerbate clinical symptoms and increase lethality. In severe cases, herd morbidity and lethality reach 48% and 30%, respectively. In contrast, herd morbidity and lethality are less than 5% and 2%, respectively (Liu, 1984). Early studies in the 1980s primarily focused on clinical symptom observation, epidemiological analysis, and virus isolation to verify presence of BVDV in yaks in high-altitude areas. Subsequent serological and molecular epidemiological investigations have demonstrated an increasing prevalence of BVDV and a high genetic diversity across different yak herds (Gong et al., 2014; Diao et al., 2020; Zhang et al., 2022; Lin et al., 2025). These findings are discussed in detail in the subsequent sections.

### 4.1 The seroprevalence for BVDV in yaks

The sero-epidemiologic studies of BVDV in yaks in China from 1980s to 2022 showed that the infection rate of BVDV in yaks was increasing, and the infection rate of yaks in Gansu and Qinghai was high (the detailed epidemiological data of BVDV in yaks are summarized in **Table 1**), so it is necessary to pay attention to the prevention and control of persistently infected cattle. A large-scale survey in more than 20 provinces and autonomous regions of China showed that the positive rate of BVDV antibody in yaks in China was 27.0% from 1980 to 1990 (Zheng et al., 1991), and the infection rates of yaks and wild yaks were relatively high. For example, the BVDV infection rates in the agricultural and pastoral areas of Yushu Prefecture, Qinghai Province, were 19.4% and 36.5% respectively (He et al., 1991). A survey of yaks in Guoluo prefecture by Dong et al. (1991) showed that BVD was mainly concentrated from April to September, and the BVD positivity rates in Maqin, Dari, and Gande counties were 11.49%, 14.81%, and 3.17%, respectively, indicating that BVD was endemic in the Guoluo region. In 1999, the study found that the seropositivity rate of yak serum in the northwest of China was 30.8%, and the antibody positivity rate of yak BVDV in Sichuan (38.46%) was higher than that in Gansu (29.41%) and Qinghai (28.00%) (Gao et al., 1999). Liu et al. (2003) found that the serum positive rates of BVDV in yaks from Kangding and Luhuo counties in Sichuan were 37.5% and 42.4% respectively, suggesting that BVDV showed signs of gradual spread in yak herds.

**Table 1:** Detailed epidemiological data of BVDV in yaks.

Year	Province	Region	Diagnostic Method	No. examined	No. Positive	Prevalence (%)	Notes	Reference
1990	No details	No details	VNT	11	3	27		(Zheng et al., 1991)
1991	Qinghai	Northwest	MNT, AGID	402 & 381	78 & 139	19.4 & 36.5	Agricultural & pastoral regions	(He et al., 1991)
1988-1990	Maqin, Qinghai	Northwest	AGID	87	10	11.49		(Dong et al., 1991)
1988-1990	Dari, Qinghai	Northwest	AGID	54	8	14.81		(Dong et al., 1991)
1988-1990	Gande, Qingai	Northwest	AGID	53	2	3.17		(Dong et al., 1991)
1999	Gansu	Northwest	MNT	102	30	29.14		(Gao et al., 1999)
1999	Qinghai	Northwest	MNT	125	35	28.00		(Gao et al., 1999)
1999	Sichuan	Southwest	MNT	39	15	38.64		(Gao et al., 1999)
2002	Kangding, Sichuan	Southwest	MNT	40	15	37.50		(Liu et al., 2003)
2002	Luhuo, Sichuan	Southwest	MNT	33	14	42.40		(Liu et al., 2003)
2010	Yushu, Qinghai	Northwest	ELISA	571	43	7.50		(Shen et al., 2011)
2011	Datong, Qinghai	Northwest	ELISA	111	26	23.42		(Han et al., 2011)
2011	Haiyan, Qinghai	Northwest	ELISA	141	28	19.86		(Han et al., 2011)
2012	Tianjun, Qinghai	Northwest	ELISA & RT-PCR	92	35	38.04		(Ye et al., 2012)
2011	Tibet	Southwest	ELISA	287	154	53.56		(Liu, 2014)
2011	Qinghai	Northwest	ELISA	262	189	72.14		(Liu, 2014)
2012	Qinghai	Northwest	ELISA	229	124	54.15		(Liu, 2014)
2012	Sichuan	Southwest	ELISA	221	100	45.25		(Liu, 2014)
2013	Tibet	Southwest	ELISA	309	103	33.33		(Liu, 2014)
2013	Sichuan	Southwest	ELISA	257	144	56.03		(Liu, 2014)
2013	Qinghai	Northwest	ELISA	2447	29	1.19	Antigen prevalence	(Fu et al., 2013)
2014-2016	Linzhi, Tibet	Southwest	ELISA	184	155	84.24		(Cheng et al., 2017)
2014-2016	Nagqu,Tibet	Southwest	ELISA	184	113	61.41		(Cheng et al., 2017)
2014-2016	Shannan, Tibet	Southwest	ELISA	92	45	48.91		(Cheng et al., 2017)
2014-2016	Lhasa	Southwest	ELISA	184	81	44.02		(Cheng et al., 2017)
2014-2016	Ngari	Southwest	ELISA	92	22	23.91		(Cheng et al., 2017)
2017	Qinghai	Northwest	ELISA	559	202	36.14		(Liu, 2017)
2018	Tibet	Southwest	ELISA	184	11	5.98		(Luo et al., 2018)
2018	Qinghai	Northwest	ELISA	390	117	30.00		(Luo et al., 2018)
2018	Gansu	Northwest	ELISA	244	43	17.62		(Luo et al., 2018)
2018	Sichuan	Southwest	ELISA	79	8	10.13		(Luo et al., 2018)
2022	Qinghai	Northwest	ELISA	231	127	54.98	Atigen prevalence (4.33)	(Luo et al., 2023)
2022	Tibet	Southwest	ELISA	86	27	31.40	Atigen prevalence (3.49)	(Luo et al., 2023)
2022	Gansu	Northwest	ELISA	87	76	87.36		(Luo et al., 2023)

AGID, agar gel immunodiffusion test; MNT, Microneutralization Test.

Ye et al. (2012) used a combination of ELISA and PCR in an epidemiologic study of BVDV in semi-wild blood yaks in Tianjun County, Qinghai, and found that the antibody positivity rate for BVDV was as high as 38.04%, with all positive samples corroborated by RT-PCR results. This further confirming the widespread transmission of BVDV in this region. In 2017, yak BVDV antibodies were detected in Nagqu, Dangxiong, Yadong of Tibet, Datong and Haibei of Qinghai, Maqu, Zhuoni and Xiahe counties of Gansu, and Hongyuan of Sichuan, and it was found that yak BVDV antibody positivity rates were 30%, 10.13%, 17.62%, and 5.98% in the Qinghai, Sichuan, Gansu, and Tibet areas, respectively (Luo et al., 2018). This result is similar to the survey results of Han et al. (2011) on yaks in Datong and Haiyan, Qinghai (positive rates of 23.42% and 19.86%), but higher than the positive rate of 7.5% for yaks in Yushu, Qinghai reported by Shen et al. (2011), and lower than the positive rate of 36.14% for yaks in Qinghai reported by Liu (2017). In addition, there are reports of higher antibody positive rates of BVDV in yaks from Tibet and Qinghai (with averages of 53.65% and 62.48% respectively, and even up to 100% in individual regions) (Gao et al., 2013). Liu (2014) found that the positive rate of BVDV antibodies in yak herds in Tibet, Qinghai and Hongyuan of Sichuan fluctuated between 33.33% and 72.14% during 2011-2013. Cheng et al. (2017) detected BVDV antibodies in yak serum from different regions of Tibet during 2014–2016 and found that the overall antibody positive rate was 48.70%. The positive rate of BVDV antibodies in yaks in Linzhi City with high breeding density (84.24%) was higher than that in Nagqu (61.41%), Shannan (48.91%), Lhasa (44.02%), Ngari (23.91%) and other places, indicating that the prevalence of BVDV has spatiotemporal heterogeneity. Fu et al. (2013) investigated 24 counties in Qinghai and showed that the positive rate of BVDV antigen was 1.19%, among which the infection rates in Hainan Prefecture (2.89%) and Huangnan Prefecture (2.21%) were relatively high, suggesting the persistently infected (PI) cattle are the key to virus transmission, because they excrete the virus throughout their lives and cannot be identified by antibody detection. Luo et al. (2023) compiled and analyzed BVDV infection in yaks on the Tibetan Plateau in the last 10 years from 2011 to 2022, and found that the average antibody detection rate of BVDV in yaks was 40.92% and the average antigen detection rate was 18.12%; they also tested yak blood samples from major yak breeding areas in Qinghai Province (Haibei Tibetan Autonomous Prefecture, Xining City, Yushu Tibetan Autonomous Prefecture), Tibet Autonomous Region (Lhasa City, Nagchu City), Sichuan Province (Aba Tibetan Autonomous Prefecture), and Gannan Tibetan Autonomous Prefecture in Gansu Province by using the blocking ELISA and double-antibody sandwich ELISA methods, and found that the total positive rate of yak BVDV antibodies was 59.82%, and the antigen positive rate was 4.42%, with Gansu yaks having a higher rate of BVDV antigen positivity (8.05%) than those in Qinghai (4.33%) and Tibet (3.49%), and they found that the percentage of persistently infected cattle was 1.55%, utilizing a protocol of sampling at 3-week intervals to re-measure BVDV antigen.

### 4.2 Co-infection of BVDV and other pathogens in yaks

A survey of 1070 yak sera in Aba Prefecture, Northwest Sichuan Province, by He et al. (2011) found that the antibody positivity rate of BVDV was as high as 44.3%, and the positivity rates of bovine coronavirus and bovine rotavirus were 84.1% and 94.4%, respectively, indicating the co-infection of multiple viral diarrhea pathogens in yaks in northwestern Sichuan is relatively common. Lv and Zhang (2014) detected serum samples of yaks from 8 counties including Dari County, Banma County, Maduo County, Jiuzhi County in Guoluo Prefecture, Zeku County, Jianzha County in Huangnan Prefecture, and Gonghe County, Tongde County in Hainan Prefecture of Qinghai Province. The results showed that the overall nucleic acid positive rate of BVDV was 21.83%, and the antibody positive rate was 23.02%; the overall nucleic acid positive rate of infectious bovine rhinotracheitis virus (IBRV) was 13.89%, and the antibody positive rate was 14.29%; and the co-infection rate of BVDV and IBRV reached 3.57%. Testing of yak fecal samples from 2016 to 2017 in Haibei Prefecture, Qinghai by Song et al. (2019) showed that the BVDV positivity rate was 51.59% in diarrheic yaks, 14.06% in healthy yaks, and the total infection rate was 26.44% (Song et al., 2019). Yang et al. (2019) found that the average infection rate of BVDV was 37.84% in Xining area, which was significantly higher than that of bovine rotavirus (27.03%) and bovine enterovirus (22.97%). The study also identified up to seven different types of mixed infections involving BVDV, bovine rotavirus, bovine enterovirus, bovine coronavirus, and bovine astrovirus. Notably, triple infections such as BVDV with bovine rotavirus and bovine enterovirus, as well as BVDV with bovine enterovirus and bovine coronavirus were observed, suggesting complex mixed infections in yaks. Similarly, Yan et al. (2019) detected 138 diarrheic yak samples in Huangzhong County, Qinghai, showing that the infection rate of BVDV was the highest (44.93%), followed by bovine enterovirus (21.74%), bovine rotavirus (8.70%), bovine astrovirus (6.52%), and bovine coronavirus (5.07%), and it is worth noting that 15.22% of the samples had a mixed infection.

Epidemiological investigations on yak diseases in the Tianshan region of Xinjiang have shown that the infection rate of BVDV in yaks is 52.0%. It often coexists with pathogens such as IBRV (81.9%), bovine coronavirus (100%), bovine parainfluenza virus (85.0%), chlamydia (44.0%), and bovine respiratory syncytial virus (20.0%), and with changes in the climate and environment, infections by environmental pathogenic bacteria such as *Pasteurella* spp. and *E. coli* also frequently occur (Yuan et al., 2015). The recent study found that the antibody positivity rate of yak pasteurellosis was 12.43%, the average antigen positivity rate of BVDV was 9.04%, and the nucleic acid positivity rate of BVDV was as high as 90.91%, suggesting that the role of BVDV in the synergistic pathogenesis of yak multi-pathogens should not be ignored (Wen et al., 2024).

### 4.3 The genotypes of BVDV in yaks

At present, the research on BVDV in yaks is relatively limited at the molecular epidemiological level. Indian researchers reported the prevalence of BVDV-1c subtype strains in yaks in the Himalayan region in 2008 (Mishra et al., 2008), most of the BVDV identified in yaks by Chinese researchers are also genotype 1, while BVDV-2 strains are usually associated with more severe clinical symptoms (such as high fever, thrombocytopenia, etc.), but studies on the prevalence of BVDV-2 in yaks in China are limited and need to be monitored more closely in the future. Yu et al. (2009) and Hu et al. (2010) analyzed the 5’ UTR, E0, and E2 genes of the Sichuan yak isolate YAK, respectively, and found that the YAK strain had a distant genetic relationship with BVDV strains in GenBank, was independent of known subtypes, and might have an independent evolutionary origin. Wang et al. (2013) further performed whole-genome sequencing on the YAK isolate of BVDV and found that the whole-genome homology with representative strains of BVDV-1 (such as CP7 and NADL) was only 69.3%~74.9%, suggesting that it might belong to a new genetic subtype. The subgenotype of the YAK isolate was not determined until 2015 when it was identified as belonging to the BVDV-1u subgenotype, a novel genetic cluster distinct from other known subgenotypes (Deng et al., 2015). Chen et al. (2018) found that the overall positive detection rate of BVDV was 19.46% by RT-PCR on the fecal samples of yaks with clinical diarrhea collected in Sichuan and Tibet in 2016, and the positive detection rate of BVDV in yaks from Tibet (27.14%) was higher than that of yaks from Sichuan (12.66%), and the viruses analyzed by sequencing were BVDV-1a and BVDV-1d. In 2011, researchers performed RT-PCR on yak blood samples from six counties in Qinghai Province and the positive rate of BVDV nucleic acid was 24%. The strains belonged to BVDV-1b, BVDV-1d and BVDV-1q sub-genotypes (Gong et al., 2014). Zhang et al. (2022) found that BVDV prquevalent in yaks in Qinghai Province mainly consisted of four subtypes, BVDV-1a, BVDV-1m, BVDV-1q and BVDV-2a, by RT-PCR amplification and sequencing analysis. Tao’s research found that BVDV in the fecal samples of diarrheic yaks in Huzhu County, Qinghai, belonged to genotype 1, with a homology of 95.7%~96.2% to the American SD-1 strain (BVDV-1a subgenotype) (Tao, 2022). Similarly, Shi et al. (2021) and Lin et al. (2025) found that the Qinghai strain (QHF4) and the Haibei isolate belonged to the BVDV-1a type by 5′UTR sequence analysis, respectively (Shi et al., 2021; Lin et al., 2025). Wang et al. (2022) isolated a cytopathogenic BVDV strain (GSTZ) from yak serum in Gansu, and determined that the nucleotide homology between its complete open reading frame and the Bega-like strain (subtype 1c) was 93.6%, respectively, by genome analysis, the GSTZ strain may have undergone long-term evolution in yaks, accumulating unique mutation sites.

## 5 Current status and prospect of prevention and control of bovine viral diarrhea in yaks

The data showed that the distribution of yaks in China accounts for 74.98% in pastoral areas and 25.02% in semi-pastoral areas. The herds have strong mobility, making it difficult to achieve comprehensive disease monitoring and vaccine immunization coverage. However, the roles of vaccine immunization and monitoring/eliminating persistently infected yaks should not be ignored. During the use of vaccines against viral diarrhea in yaks, other vaccines can also be rationally applied in combination with the epidemiological background of other diseases in local yaks to establish an immune barrier. In the early 1980s, researchers from the Department of Animal Husbandry and Veterinary Medicine of Southwest University for Nationalities explored the cross-immunization effect of classical swine fever attenuated vaccine against viral diarrhea in yaks, and achieved certain results. Ji et al. (1995) reported that more than 40,000 yaks in Qinghai Province were immunized with the rabbit-adapted attenuated vaccine against classical swine fever, and the mortality rate of the immunized yak herd (0.27%) was significantly lower than that of the non-immunized group (3.28%). Liu et al. (2003) found that the use of rabbit-adapted tissue attenuated vaccine for controlling BVD was safe and effective at a dosage of 3 immune units per yak calf and 6~10 units for adult yaks. Liu et al. (2013) conducted a field immunization trial in yaks using a BVD inactivated vaccine and a rabbit-adapted classical swine fever attenuated vaccine, and the results indicated that both vaccines could induce antibody production, and the positive antibody rate peaked on day 14, and the overall antibody level of the BVD vaccine was significantly higher than that of the classical swine fever vaccine. Luo et al. (2023) conducted a study on the protection period of a BVD inactivated vaccine in yaks and found that the antibody titer peaked at 55.3 days after immunization with the commercial inactivated vaccine. Zhang et al. (2024) carried out immunization studies on yaks using inactivated vaccines against BVD, bovine paratyphoid, and *Pasteurella multocida*, and found that the antibody titer of yaks immunized with the inactivated BVD vaccine peaked at 60 days and could be maintained for 6 months. In contrast, the antibody titers of yaks vaccinated with inactivated vaccines against bovine paratyphoid and *P. multocida* were relatively low. These studies indicate that the previous passive situation of no special vaccines available for the prevention and control of bovine viral diarrhea in yaks is expected to be reversed.

China has made important progress in the pathogen identification, molecular epidemiology, vaccine development, and comprehensive prevention and control of BVDV. However, research on the molecular characteristics, pathogenic mechanisms, immune responses, and antigenic differences from existing vaccines of BVDV strains derived from yaks is relatively weak. In addition, as a special breed of yak, the characteristics of its immune system in response to viruses and vaccines may be different from those of other cattle breeds, which need to be further investigated. In China, yaks are primarily distributed across the Qinghai-Tibet Plateau and are mainly raised through pastoral grazing systems, similar to the husbandry practices for sheep. In yak-farming provinces, dairy cattle populations are relatively small and predominantly maintained under intensive farming systems. In addition, approximately 30,000 wild yaks still persist as wildlife on the Qinghai-Tibet Plateau. Therefore, the current risk of BVDV transmission through direct contact between cattle and yaks on the Qinghai-Tibet Plateau may be lower than the transmission risks between yaks and sheep, or between domestic and wild yaks. Recent paired serological studies in Tibet (testing yaks at 3-week intervals) have detected BVDV antigens, confirming the establishment of persistent BVDV infections in yaks. This finding suggests that transmission cycles of BVDV can be likely maintained within yak populations, but whether yaks can act as true reservoirs remains unclear. In the prevention and control of BVD, there are some successful experiences to learn from in BVD prevention and control, generally in high BVDV endemic areas, vaccination should be the main approach combined with screening and culling of persistently infected animals; in areas with low prevalence of BVDV, emphasis can be placed on strengthening biosecurity measures and monitoring systems. Prevention and control strategies in different regions can be appropriately adjusted according to the epidemiological background data of yak BVD, which should be suitable for the characteristics of plateau pastoral areas. However, regardless of the strategy adopted, long-term persistence and wide coverage are the keys to achieving prevention and control effectiveness. Due to the multiple transmission routes of bovine viral diarrhea, such as vertical transmission, direct contact, and indirect transmission through pollutants, and the fact that some infected cattle are in a recessive virus-carrying state, single prevention and control measures are difficult to be effective in the prevention and control of yak BVD.

It is necessary to fully combine traditional grazing methods with modern farming concepts, taking into account multiple links such as biosecurity and disinfection, feeding management and nutritional control and other aspects, adhering to the basic principles of the prevention-oriented and prevention and control combination, to build a systematic “Five-dimensional Integration” comprehensive prevention and control model, including vaccine breakthrough, precise monitoring, dynamic early warning, population purification, and active prevention.

In terms of vaccine breakthrough, current domestic BVD and BVD-IBR vaccines demonstrate potential for disease control, with researchers particularly focused on evaluating the immune response of existing inactivated BVD vaccines in yaks (Zhang et al., 2024), though further studies are required to assess their immunization efficacy and protective coverage against circulating BVD strains in yak populations. Future vaccine development strategies could benefit from considering broader-spectrum formulations, including combination BVDV vaccines covering both BVDV-1 and BVDV-2 strains, as well as polyvalent vaccines combining BVDV with other major yak pathogens such as *Pasteurella* spp., *E. coli*, bovine rotavirus, bovine enterovirus, and bovine coronavirus, along with various combinations targeting different pathogen profiles.

In terms of precise monitoring, it mainly relies on viral antigen screening or molecular diagnostic techniques to actively target the PI yaks with BVDV and cut off the virus transmission chain. In the current epidemiological studies on BVDV from yaks in Qinghai and Tibet, there have been reports of antigen-positive cases, especially persistent infections, leading to the continuous circulation of the virus in cattle herds, so culling PI yaks can effectively block the transmission chain. Meanwhile, regular detection should be conducted to exclude the introduction of wild-type strains during livestock trading. In addition, it is necessary to assess the implementation difficulties and some economic losses caused by the scattered breeding of yak herds. With the promotion of modern breeding management concepts, farmers and breeders should establish a reasonable yak trading mechanism, optimize the herd structure, and improve the elimination mechanism oriented by improving breeding efficiency. In terms of population purification, pathogen purification can be carried out at key links in breeding livestock farms to strengthen the purification of breeding sources. China’s Health Standards for Breeding Animals has also listed the absence of BVD as one of the health standards for breeding cattle, which is of great significance for ensuring the genetic quality of yaks, reducing economic losses, collaboratively preventing and controlling other cattle diseases, and enhancing market competitiveness. The relevant studies also reported the purification measures for BVD in cattle farms, including biosecurity and introduction safety, background investigation, immunization and monitoring, breeding management, PI cattle culling, which are worthy of promotion and reference in yak farms with corresponding infrastructure and management level (Dong, 2022; DB22/T 3521.2-2023, 2023).

In terms of dynamic early warning and proactive prevention, passive monitoring can be carried out by combining the diagnosis of suspected BVD cases in yaks by official veterinary technicians and farmers, and in conjunction with the National Animal Disease Surveillance and Epidemiological Investigation Plan, the epidemic situation of BVD in yak herds should be scientifically evaluated in active monitoring to predict the evolutionary trends of the virus, which requires shifting the prevention and control focus forward, controlling potential BVDV contamination in biological products such as sera, cell cultures, and veterinary vaccines. It is also crucial to promote the transformation of prevention and control concepts among breeding farmers, shifting from passive response to BVD to systematic proactive prevention and control. The relevant studies also showed that some comprehensive measures, such as antibacterial, antiviral, gastrointestinal function regulation, acidosis correction, fluid and vitamin supplementation for controlling BVD, and these measures have practical value for alleviating the clinical symptoms of yak diarrhea and reducing the mortality rate (Li and Jiang, 2007; Zhao et al., 2008; Lang, 2024; Ma et al., 2024).
